# Prediction of damage intensity to masonry residential buildings with convolutional neural network and support vector machine

**DOI:** 10.1038/s41598-024-66466-3

**Published:** 2024-07-15

**Authors:** Adrian Jędrzejczyk, Karol Firek, Janusz Rusek, Umberto Alibrandi

**Affiliations:** 1 Faculty of Geo-Data Science, Geodesy and Environmental Engineering, AGH University, Kraków, Poland; 2 Department of Civil and Architectural Engineering, Aarhus University, Aarhus, Denmark

**Keywords:** Convolutional neural network, Support vector machine, Damage intensity, Damage intensity prediction, Masonry buildings, Residential buildings, Civil engineering, Computational science

## Abstract

During their life cycle, buildings are subjected to damage that reduces their performance and can pose a significant threat to structural safety. This paper presents the results of research into the creation of a model for predicting damage intensity of buildings located in mining terrains. The basis for the research was a database of technical and mining impact data for 185 masonry residential buildings. The intensity of damage to buildings was negligible and ranged from 0 to 6%. The Convolutional Neural Network (CNN) methodology was used to create the model. The Support Vector Machine (SVM) methodology, which is commonly used for analysis of this type of issue, was used for comparisons. The resulting models were evaluated by comparing parameters such as accuracy, precision, recall, and *F*_1_ score. The comparisons revealed only minor differences between the models. Despite the small range of damage intensity, the models created were able to achieve prediction results of around 80%. The SVM model had better results for training set accuracy, while the CNN model achieved higher values for *F*_1_ score and average precision for the test set. The results obtained justify the adoption of the CNN methodology as effective in the context of predicting the damage intensity of masonry residential buildings located in mining terrains.

## Introduction

Residential buildings are the most numerous group of buildings in Poland. According to the Central Statistical Office, there are nearly 6.9 million of them^1^. In 2022, more than 110,000 new residential buildings were approved for use, of which as many as 98.5% were built using traditional (masonry) technology^2^. Therefore, this group is a very important one from a technical and social point of view.

Masonry residential buildings, like other structures, are exposed to damage during their life cycle. Analysed in terms of structural mechanics, this damage is a process initiated when the allowable level of elastic deformation potential energy stored in a given structural element is exceeded^3,4^. This is a major criterion in the design of building structures and determines the maintenance of safety and acceptable levels of performance values^5^.

Damage to buildings can be caused by a wide range of construction and environmental factors [e.g.^6^]. The construction factors may include, but are not limited to, defects in workmanship, improper use, aging of materials, or poor maintenance management^7^. Among the environmental factors, it is important to point out the mining-induced near-surface rock destruction processes that cause tremors and largescale deformation of the ground surface^8^. These impacts induce kinematic loads transmitted through the ground to the building structures, and this in turn can have a negative impact on the structures^9–12^ and initiate the damage process. Most often, the initiation of the damage process depends not on one, but on many factors simultaneously. This means that the problem regarding the assessment of the causes of damage is highly multifactorial and, from an analytical point of view, difficult to describe.

Most of the research to date related to damage to buildings and their components has been (and still is) carried out using statistical methods and conventional machine learning classifiers. Among these, Support Vector Machines, which are stable and proven classifiers, should be singled out^13,14^. Less frequently, Probabilistic Neural Networks or Random Forests are used in this research^15,16^. In recent years, there has been a dynamic development of deep learning algorithms used in the field of Computer Vision. They make it possible to achieve high accuracy in, for example, classification tasks. One method that performs well in research related to image recognition is CNN^17–20^. In Computer Vision Tasks, information is conveyed by the pixels that make up the image. To date, in the building industry, the CNN method has found application in detecting changes to objects in satellite or aerial images^21–23^. In addition, an increasing number of articles have been found in recent years on the detection of surface damage to the building envelope^24^ and other structures^25^. Also noteworthy is the research into the use of acoustic techniques in the field of structural monitoring using the CNN method^26,27^.

Based on our own experience using previous research, the aim of this study was defined, which was to create a model for the prediction of damage to masonry residential buildings using the Convolutional Neural Network (CNN) method from the field of deep learning. The application of a method from the field of image analysis required the preprocessing of numerical data into a quasi-image. As a result of the study, the suitability of the selected CNN method for damage prediction was evaluated. The Support Vector Machine (SVM) method, proven in issues of this type, was adopted for comparison.

From the point of view of building users, every damage is significant, and its repair generates costs. This is why predicting even small changes in the intensity of damage to buildings is so important, especially as most of the identified damage has a small extent. Most of the research to date is concerned with predicting damage categories with a more widely range. This paper presents the results of close-range damage intensity prediction using two methods. “Materials” section presents the database and describes the preparation of the data for analysis. “Methods” section presents the methods used in the study. “Results and discussion” section presents the architecture of the created CNN, the hyperparameters of the SVM model, the obtained results and the discussion. “Conclusions” section concludes this article. Created models have been tested for existing buildings that have been subjected to mining impacts during their life cycle. The methodology used can be considered in terms of predicting the damage intensity of large groups of buildings that are subjected to anthropogenic environmental influences that can induce damage.

## Materials

### Characteristics of the database

The object of the research described in this paper was a set of 185 residential buildings of traditional masonry structure, located in a mining terrain, which had not yet undergone significant interventions in the form of repairs and preventive protection. An inventory of the architectural-structural and technical condition was carried out for these buildings. The obtained information was stored in a database. This included data on the geometry and construction of the building, as well as other technical data which affect the building's resistance to mining impacts, such as age and maintenance quality.

The database created included buildings up to 68 years old (32 years on average). The buildings range in length from 7 to 24m. Simple-plan buildings predominate (50%), with disjointed shape buildings accounting for 28% and heavily disjointed shape buildings accounting for 22%. 137 buildings were founded at a fixed level. The foundations of most of the buildings were made of concrete (58%), less often of reinforced concrete. Basement walls were made of monolithic concrete (58%), less frequently of masonry (25%) or concrete blocks (17%). The load-bearing walls of the ground and upper floors were mainly made of cellular concrete blocks or silicates (76%), less frequently of cinder blocks (18%) or clay bricks (6%). The analysed group of buildings is characterised by a large diversity in ceiling construction. The first ceiling was most often made as a dense-rib or monolithic reinforced concrete floor, prefabricated or on steel beams (79%), less frequently as a timber (15%) or brick infill floor (6%). In most cases (57%), continuous perimeter beams were used at the ceiling support on the walls. The covering in all buildings was made as a timber roof truss.

All the buildings analysed were exposed to mining impacts during their life cycle. Based on information obtained from the mining plant, the values of continuous deformation and tremor indicators were determined. Preliminary analyses showed that, in the analysed area, tensile *ε*^+^ and compressive *ε*^*-*^ horizontal ground strains are important in the context of building technical wear.

As a result of preliminary analyses, it was concluded that the indicators characterising the tremor impacts do not demonstrate a statistically significant influence on the damage intensity and technical condition of the analysed buildings. Therefore, the impacts of mining tremors on buildings were not further considered in this paper.

### Determination of damage intensity index values for individual buildings

The methodology used to determine the damage intensity index was that presented in papers^28,29^. According to this, Partial Least Squares Regression (PLSR) was used to determine a generalised damage intensity index for the whole building, based on the results of the iterative NIPALS (Nonlinear Partial Least Squares) algorithm^30–34^.

Using the methodology described in^28^ and applying the above-described algorithm, it was determined that the description of the generalised damage index *w*_*u*_ for the analysed groups of buildings are linear combinations of the damage indices of the structural and finishing elements *w*_*ui*_ with the general form of the formula:

1 $$w_{u} = \mathop \sum \limits_{i = 1}^{n} a_{i} w_{ui}$$

where $a_{i}$ directional coefficients of the linear combination of components occurring at individual damage indicators determined by the PLSR method, $w_{ui}$ the value of the damage intensity indicators of the individual building elements.

Based on the determined directional coefficients *a*_*i*_ and the values of the damage intensity index *w*_*ui*_ for each building in the database, the value of the generalised damage intensity index *w*_*u*_ was determined. The average value of the *w*_*u*_ index for the analysed buildings was 2.7%.

### Data clustering

The predominance of qualitative variables over quantitative variables makes it possible to state that the analysed problem is a non-linear issue. Therefore, classification methods were chosen to predict the damage intensity of buildings.

In both regression and classification tasks, it is very important to prepare the data for analysis. In the case of regression, proper ranking (gradation) of the data is important. In the case of classification, the main problem may occur with continuous variables, which have the potential to convey more information than categorised variables; however, too high a variety of values significantly increases the number of iterations and increases computation time. In turn, the lack of clearly defined boundaries (appropriate clustering) can lead to inappropriate results.

In this paper, the K-means method was used to cluster continuous variables. Additional observations of mean values and analysis of the normal distribution curve allowed the method to be used to appropriately rank the variables.

The analyses were carried out for a group of 185 unrenovated and unprotected (against mining impacts) buildings. For this reason, indicators for renovation and protection were not included in the study. Indicators of mining impacts were also excluded from the K-means analysis, for which the well-known division into mining terrain categories was used (Table 1), which will be denoted by Arabic numerals in the following section.

**Table 1:** Categories of horizontal deformation of the mining terrain^35^.

Category	0	I	II	III	IV	V
Horizontal deformation *ε* [mm/m]	∣*ε*∣ ≤ 0.3	0.3 < ∣*ε*∣ ≤ 1.5	1.5 < ∣*ε*∣ ≤ 3	3 < ∣*ε*∣ ≤ 6	6 < ∣*ε*∣ ≤ 9	∣*ε*∣ > 9

The parameters selected for further analysis and the number of compartments (clusters) into which they were divided are shown in Table 2.

**Table 2:** Summary of parameters selected for analysis.

Variable type	Parameter	Number of clusters (categories)
Geometry	Length	5
	Building shell shape	2
	Horizontal projection shape	3
	Foundation level	2
Construction	Foundations	2
	Basement walls	3
	Upper walls	3
	First ceiling	5
	upper ceilings	5
	Beams	2
Other data	Age	10
	Maintenance quality	2
Mining impacts	*ε*^+^	4
	*ε*^*-*^	4
Damage	*w*_*u*_	3

Compared to the other variables, damage, represented by the *w*_*u*_ intensity index, is a predictive variable. The damage intensity of the 185 unrenovated and unprotected buildings stored in the database is between 0 and 6%, which means negligible damage^28,29^. This state of affairs led to the need to establish a more detailed distribution to explore the possibility of damage prediction on the dataset. A K-means cluster analysis found that a division into three categories would be optimal (Table 3).

**Table 3:** Division of the damage intensity index *w*_*u*_ into clusters with established categories.

Parameter	Value	Category
*w*_*u*_ [%]	≤ 1.7	1
	1.7 to 3.5	2
	> 3.5	3

Subsequently, the data summarised in Table 2, with the exception of the *w*_*u*_ damage index, were transformed into quasi-images.

### Data transformation into quasi-images

**Figure 1:**
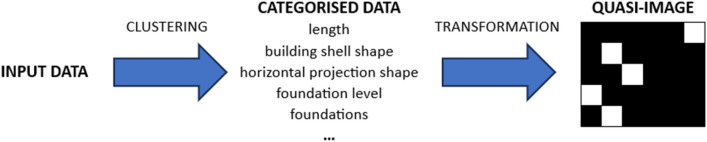
Data preparation scheme for CNN.

In the present study, data collected in numerical form were used for the research. Therefore, they required appropriate preparation before implementing the CNN. First, the input data was clustered (cf. “Data clustering” section). The resulting categorised data were transformed into quasi-images (Fig. 1).

**Figure 2:**
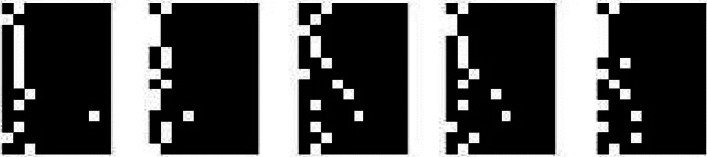
Examples of monochrome quasi-images resulting from data transformation.

The transformation resulted in monochrome quasi-images, in which the position of the white pixel in the row informs us of the cluster(category) values obtained for the subsequent parameters (cf. Table 2). Examples of the obtained images are shown below (Fig. 2).

Each row represents a horizontal section of the created image with a height of one pixel. The order in which the numerical data are stored in the image follows the order of the parameters listed in Table 2. The quasi-images were used in further analyses.

## Methods

### Convolutional neural network (CNN)

**Figure 3:**
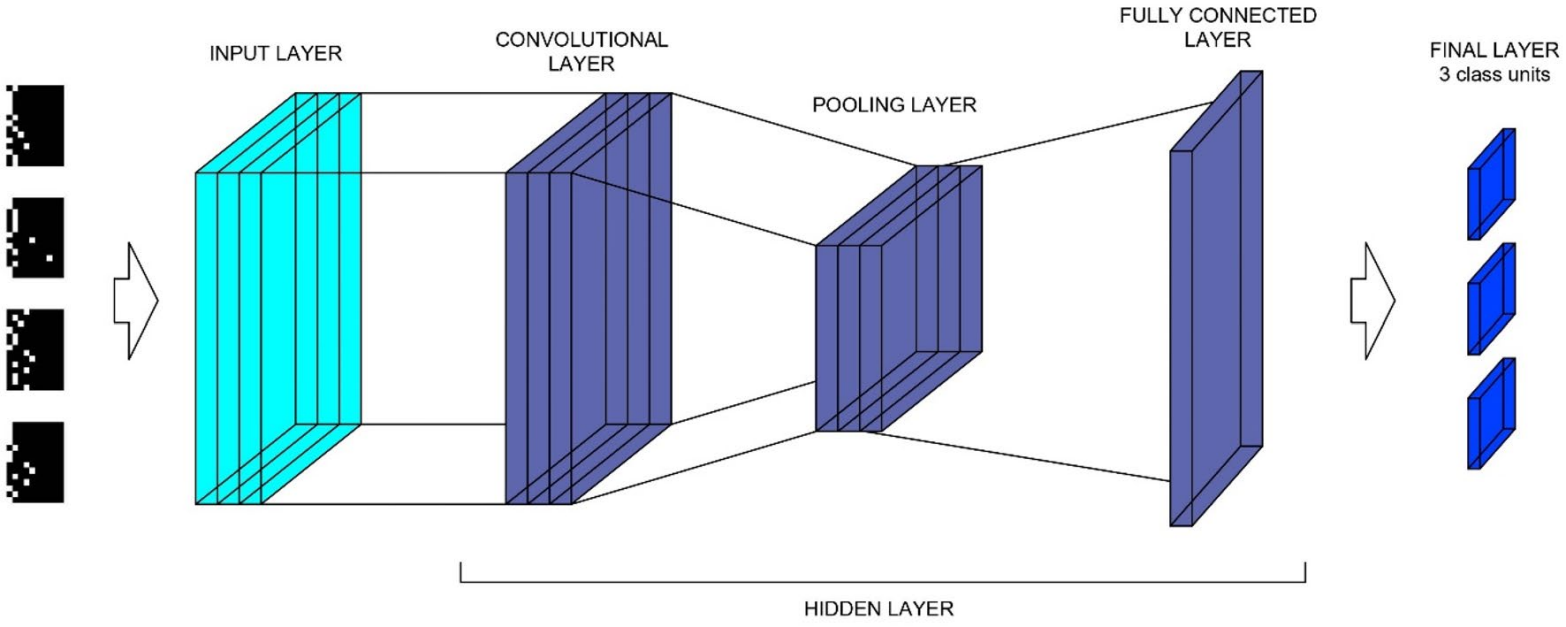
General scheme of a Convolutional Neural Network.

Starting to solve problems using deep neural networks, it is necessary to establish the type and architecture of the neural network. The network architecture created should be optimal, i.e. handle the input data in the best possible way. Figure 3 shows the general scheme used in CNN networks. Initially, data in the form of images are loaded into the network. Subsequently, the data are fed into the hidden layer. In neural networks, there can be many hidden layers. In CNN networks, there are three main types of hidden layers: convolutional, pooling and fully connected^36^. The network learns optimal filters through back propagation and gradient descent. At the output of the network (final layer), a prediction is made.

The basic hidden layer in CNN is the **convolutional layer**. It consists of a set of learning filters (kernels) that extract differences between images. The filters are small matrices (e.g. 2 × 2 px.) that slide through the entire input volume. The sliding of the filters results in two-dimensional outputs that, when combined, form an activation map^14,36^. The specification of hyperparameters such as padding, strides, size, and number of filters (neurons) in a layer, is up to the user. The values in the filters are selected and optimised during the network learning process.

In high-resolution images, neighbouring pixels usually have similar values. Thus, it can be concluded that many pixels convey information about the same element in the image at the same time. Too much information about the same element creates 'noise' and is unnecessary. To remove the 'noise', the **pooling layer** is used that reduces the dimensions of the feature maps on the plane (down-sampling). The specification of hyperparameters such as padding, strides and kernel (filter) size, in this case, is also up to the user. The most common types of pooling layers include maximum, minimum or medium pooling^14,37^.

The fully connected layer creates connections with each neuron from the previous layer. In the learning process, these connections are assigned weights, which the CNN then uses in determining the probability of assignment to the resulting class. Finally, this layer has the same number of output nodes and classes^14,38^.

The correct performance of the network, in addition to the above-described layers, also depends on the applied non-linear activation functions that form the decision boundaries, which is of major importance in the correct prediction of the results^37^. In addition to the network architecture described above, the learning algorithm also plays a key role in the performance of the CNN.

### Support vector machine SVM

To compare the results achieved by the CNN network, SVM was used, which is one of the most reliable classification and regression algorithms in multiple applications fields.

**Figure 4:**
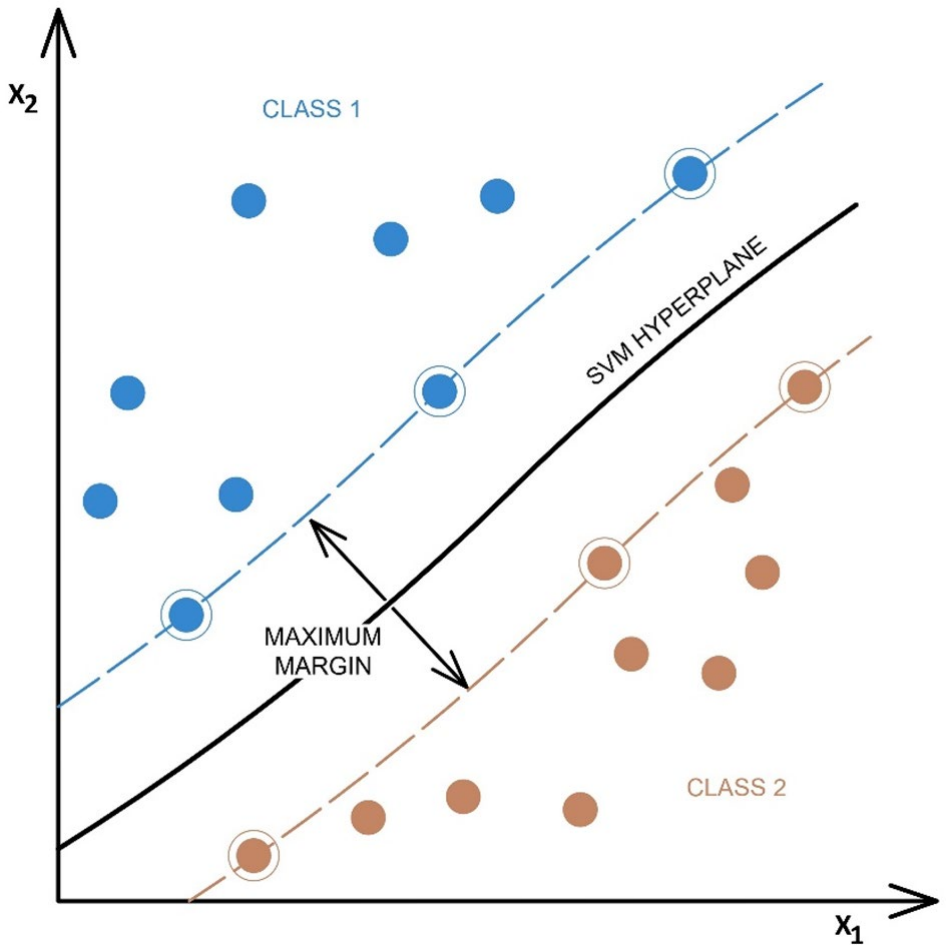
Example of a binary SVM classifier with best-fit hyperplane^7^.

Currently, an SVM classifier can be used to solve problems of classifying data into two or more classes. The data are represented by multidimensional vectors, each of which belongs to one of the classes. The data are separated using a decision boundary (hyperplane). During learning, the SVM algorithm searches for a hyperplane separating classes of points with the maximum possible distance (separation margin) between them (Fig. 4). A hyperplane fitted in this way will better generalise and classify the test data^39^.

The most commonly used types of kernel functions in SVM are Gaussian, radial, sigmoidal or polynomial representations. With these functions, both regression and classification tasks are performed^40^. In addition to the mentioned function types, a linear description of the hyperplane can be used, which is usually inefficient. The SVM uses Cover's theorem, which involves increasing the dimension of the feature space with regard to the projected x-patterns^41^. This leads to a transformation *φ(x)*, that results in a hyperplane, with the following form:

2 $${\text{y}}\left( {\mathbf{x}} \right) = {\mathbf{w}}^{{\text{T}}} \varphi \left( {\mathbf{x}} \right) + {\text{b}} = 0$$

where ${\text{x}} \in {\text{R}}^{{\text{n}}}$ input data vector in the n-dimensional space, $\upvarphi :{\text{R}}^{{\text{n}}} \to {\text{R}}^{{{\text{n}}_{{\text{h}}} }}$ certain transformation converting raw input data into the feature space, **w**^T^ weights vector, b bias (free component).

The transformation $\upvarphi :{\text{R}}^{{\text{n}}} \to {\text{R}}^{{{\text{n}}_{{\text{h}}} }}$ is given in an implicit way, and the key impact on its form is the kernel function. Finally the SVM classifier, according to^42^, can be written as:

3 $${\text{y}}\left( {\mathbf{x}} \right) = \mathop \sum \limits_{{{\text{k}} = 1}}^{{{\text{N}}_{{{\text{sv}}}} }} \upalpha _{{\text{k}}} {\text{d}}_{{\text{k}}} {\text{K}}\left( {{\mathbf{x}},{\mathbf{x}}_{{\text{k}}} } \right) + {\text{b}}$$

In the given Eq. (3), there is a factor K, called the system kernel, which is predetermined explicitly and is the result of a combination of implicit functions $\upvarphi$:

4 $${\text{K}}\left( {{\mathbf{x}}_{{\text{k}}} ,{\mathbf{x}}_{{\text{j}}} } \right) = \varphi \left( {{\mathbf{x}}_{{\text{k}}} } \right)\varphi \left( {{\mathbf{x}}_{{\text{j}}} } \right)$$

The form of the system kernel is selected arbitrarily from among the functions meeting the conditions of Mercer's theorem^43^.

An important step in building an SVM model for classification tasks is to determine the values of parameters such as σ and γ. Both parameters determine the learning process. The regularisation constant σ also called box constraints occurs in the loss function. Whereas γ determines the curvature of the kernel function (5).

5 $${\text{K}}\left( {{\mathbf{x}}_{{\text{k}}} ,{\mathbf{x}}_{{\text{j}}} } \right) = {\text{exp}}\left( { - \frac{{\left( {{\mathbf{x}}_{{\text{k}}} - {\mathbf{x}}_{{\text{j}}} } \right)^{2} }}{{\gamma^{2} }}} \right) = {\text{exp}}\left( { - \sigma \left( {{\mathbf{x}}_{{\text{k}}} - {\mathbf{x}}_{{\text{j}}} } \right)^{2} } \right)$$

In this paper, the optimal parameter values were ascertained using the Bayesian optimisation method^44^.

## Results and discussion

The implementation of the classification tasks requires, before the research, the division of the data into a training set and a test set. Following the principles proposed in the literature^45^, the set of 185 unrenovated and unprotected buildings adopted for the study was randomly divided in a 75:25 ratio into a training set and a test set. This yielded 139 cases for the training set and 46 cases for the test set.

In order to quantitatively assess the obtained models and enable them to be compared with each other, the parameters describing classification accuracy were collected in a confusion matrix. The basic form of this matrix for the binary classifier is shown in Table 4.

**Table 4:** Confusion matrix for a binary classifier^46^.

		Actual	
		Positive	Negative
Predicted	Positive	True Positive *TP*	False Positive *FP*
	Negative	False Negative *FN*	True Negative *TN*

The main measure for evaluating a classifier is the classification accuracy ACC^47^, which is calculated using Eq. (6) and represents the percentage of the classifier's predictions.

6 $$ACC = { }\frac{TP + TN}{{TP + FP + FN + TN}}$$

In addition to accuracy, the parameters that were used to characterise the classification model were:

- precision *PPV*:
  7 $$PPV = { }\frac{TP}{{TP + FP}}$$
- recall *TPR*:
  8 $$TPR = { }\frac{TN}{{FN + TN}}$$
- harmonic average called the *F*_1_ score [52]:
  9 $$F_{1} = { }2{*}\frac{PPV*TPR}{{PPV + TPR}}$$

In order to descriptively evaluate the results obtained, qualitative criteria were introduced depending on the accuracy of the built models. Based on the author’s experience, for precision, recall and accuracy, the following criteria were adopted:

-  > 85%—high,
- 75 ÷ 85%—good,
- 65 ÷ 75%—acceptable,
- 55 ÷ 65%—average,
-  < 55%—low.

Besides assessing accuracy, it is very important to assess the generalisation ability of the model^39^, which consists of comparing the classification accuracy for the learning and test sets with each other. A small difference in accuracy between the sets indicates the ability of the model to predict the correct response for cases that are not involved in the learning process. Significantly higher accuracy for the learning set may indicate overlearning of the model. Based on the author’s experience, the following qualitative criteria for assessing generalisation ability due to differences in accuracy were adopted:

-  < 5%—high,
- 5 ÷ 10%—good,
- 10 ÷ 15%—average,
-  > 15%—low.

### Convolutional neural network

**Figure 5:**
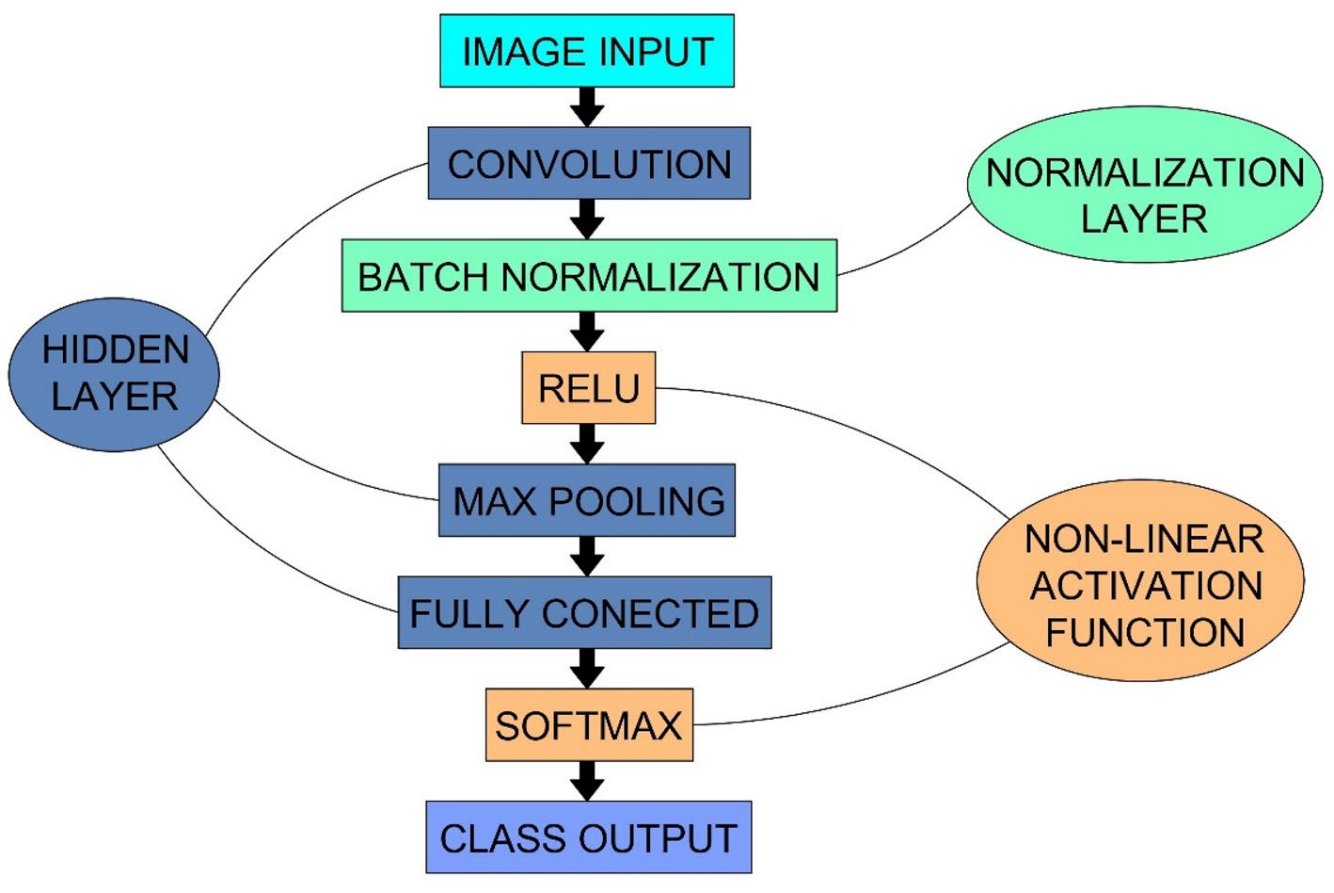
Architecture of the created CNN network.

CNNs offer great opportunities to create a complex network architecture^47^. However, the architecture expansion does not always lead to better performance. The creation of a network with a simple architecture was sufficient, with a schematic diagram shown in Fig. 5.

Figure 5 presents a scheme of the network architecture created for the defined task. Compared to Fig. 3, inside the created network, a normalisation layer (batch normalisation) and non-linear activation functions (relu and softmax) appeared. First in the diagram is, the image input layer, which was used to load the data. The next layer (first hidden) that was used in the network is the convolution layer, for which filters of 1 × 3 pixels with 8 neurons per layer were applied. Then, a normalisation layer (batch normalisation) and a rectified linear activation (relu) function were applied to achieve the necessary non-linearity. To reduce noise, down-sampling by using a pooling layer (max pooling) was performed. For this, filters of 1 × 3 pixels were used. A third (last) hidden layer (fully conected) was used to create connections within the network. For the final prediction, the softmax function and the class output layer were used. By passing the softmax, the probability was determined, while the output layer identified the predicted classes.

**Figure 6:**
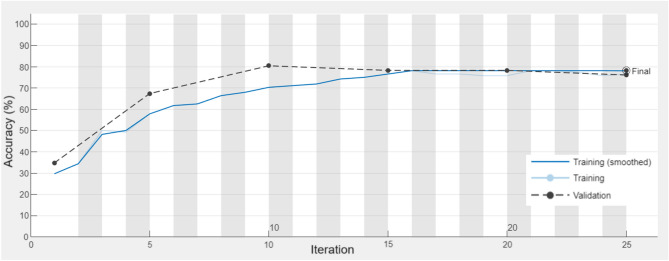
The learning process of the created CNN network.

The proper operation of the network is ensured by its architecture together with the learning algorithm. In this case, it was found that Stochastic Gradient Descent with Momentum (SGDM) algorithm proved to be the most accurate for the given task. The learning process using the SGDM algorithm is presented in Fig. 6.

Figure 6 presents the classification results during the training and testing process of the created network. The solid line shows the results for the training set, while the dashed line shows the results for the test set. In the initial learning phase, the classification accuracy for the training and test set was over 30% and increased with subsequent iterations, reaching an accuracy of close to 80% after 15 iterations. Detailed classification accuracy results for the created model are shown in Table 5.

**Table 5:**
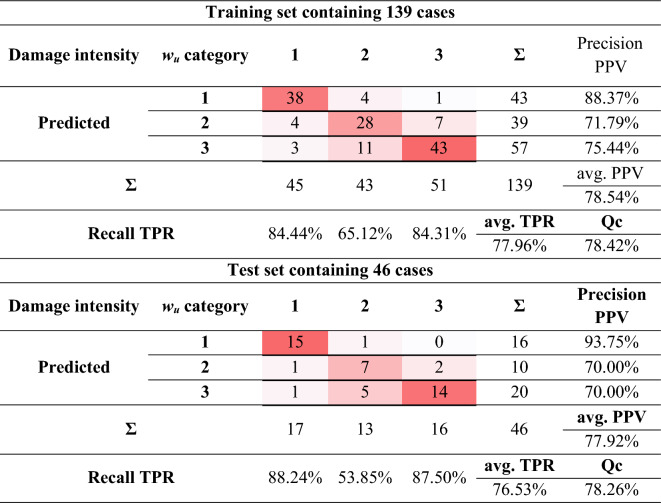
Confusion matrix of the CNN classifier.

The created model is characterised by good classifier accuracy for the training set *ACC* = 78.42% and the test set *ACC* = 78.26%. The small difference in accuracy of *∆ACC* = 0.16% confirms the high generalisation ability of the classifier.

For the training set, the following degree of correctness of the classified patterns was concluded:

- For classifying the data into 1st damage category—a high level of precision *PPV* = 88.37% and a good level of recall *TPR* = 84.44% obtained were obtained,
- For classifying the data into 2nd damage category—an acceptable level of precision *PPV* = 71.79% and recall *TPR* = 65.12% were obtained,
- For classifying the data into 3rd damage category—a good level of precision *PPV* = 75.44% and recall *TPR* = 84.31% were obtained.

For the test set, the following degree of correctness of the classified patterns was concluded:

- For classifying the data into 1st damage category—a high level of precision *PPV* = 93.75% and recall *TPR* = 88.24% were obtained,
- For classifying the data into 2nd damage category—an acceptable level of precision *PPV* = 70.00% and a low level of recall *TPR* = 53.85% were obtained,
- For classifying the data into 3rd damage category—an acceptable level of precision *PPV* = 70.00% and a high level of recall *TPR* = 87.50% were obtained.

The differences in the degree of classified patterns between the training and test set are negligible, within 6%. The exceptions are the values obtained for recall *∆TPR* = 11.27% for the 2nd damage category. The average precision and recall are at a similar level.

### Support vector machine

Following the established course of analysis described in Sect. 3.2, model construction began with the determination of the optimal σ and γ hyperparameters. For this objective, we used Bayesian optimisation using cross-validation^48^. Radial basis functions (RBF) were adopted as the kernel function of the model, which presented slightly better accuracy results than Gaussian functions. The obtained results are shown in Table 6.

**Table 6:** Summary of basic parameters characterising the SVM model.

Optimisation method	Model parameters			
	Regularisation constant σ	Function width γ	Kernel function	Number of Support Vectors
Bayesian optimisation	0.69	4.07	RBF	90

The results presented in the Table 6 show that the size of the SVM classifier structure, i.e. the number of support vectors (90), was reduced by 35% compared to the size of the training set (139). This is due to the regularisation that occurs in this type of approach. The significant reduction in the number of learning patterns may indicate good generalisation properties of the model.

An evaluation of the classification accuracy of the created model was made by using a confusion matrix, which is shown in Table 7.

**Table 7:**
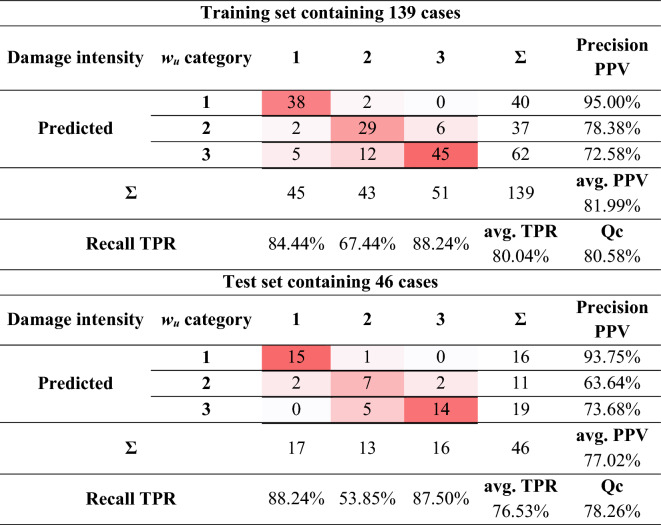
Confusion matrix of the SVM classifier.

The created model is characterised by good classifier accuracy for the training set *ACC* = 80.58% and the test set *ACC* = 78.26%. The small difference in accuracy of *∆ACC* = 2.32% confirms the high generalisation ability of the classifier.

For the training set, the following degree of correctness of the classified patterns was concluded:

- For classifying the data into 1st damage category—a high level of precision *PPV* = 95.00% and a good level of recall *TPR* = 84.44% were obtained,
- For classifying the data into 2nd damage category—a good level of precision *PPV* = 78.38% and an acceptable level of recall *TPR* = 67.44% were obtained,
- For classifying the data into 3rd damage category—an acceptable level of precision *PPV* = 72.58% and a high level of recall *TPR* = 88.24% were obtained.

For the test set, the following degree of correctness of the classified patterns was concluded:

- For classifying the data into 1st damage category—a high level of precision *PPV* = 93.75% and recall *TPR* = 88.24% were obtained,
- For classifying the data into 2nd damage category—an average level of precision *PPV* = 63.64% and a low level of recall *TPR* = 53.85% were obtained,
- For classifying the data into 3rd damage category—an acceptable level of precision *PPV* = 73.68% and a high level of recall *TPR* = 87.50% were obtained.

The differences in the degree of classified patterns between the training and test set are negligible, within 4%. The exceptions are the values obtained for precision *∆PPV* = 14.74% and recall *∆TPR* = 13.59% for the 2nd damage category. The average precision and recall are at a similar level.

### Results comparison

**Figure 7:**
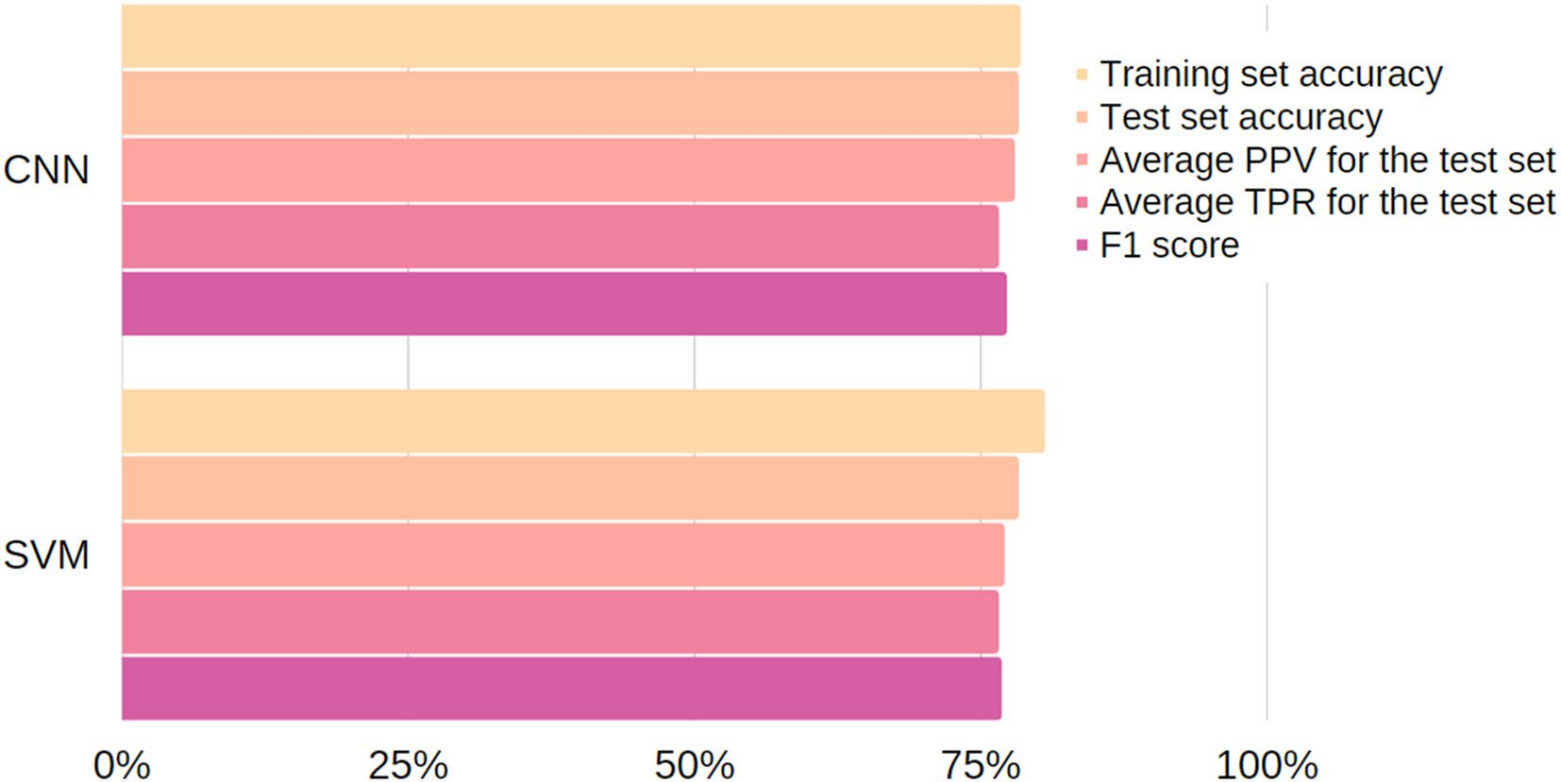
Graphical overview parameters of the compared models.

On the basis of the conducted analyses and the determined parameters of the individual models, the results were compared. The main evaluation criterion was the classification accuracy of the test set. Due to the similar (high) generalisation abilities of the compared models, these were not taken into account. Measures such as precision *PPV*, recall *TPR* and *F*_1_ score were used for comparison. The parameters of the models are shown in Table 8 and Fig. 7.

**Table 8:** Comparison of model parameters.

Method	Accuracy		Test set		*F*_1_ score (%)
	Training set (%)	Test set (%)	Average PPV (%)	Average TPR (%)	
CNN	78.42	78.26	77.92	76.53	77.22
SVM	80.58	78.26	77.02	76.53	76.77

From Table 8 and Fig. 7, it was concluded that the obtained results express similar values, with the exception that the classification accuracy for the training set reached a higher value for the SVM method. In contrast, the average precision *PPV* and *F*_1_ score reached higher values for the CNN method. A comparison of the models does not make it clear which method performed better. However, given the obtained results, it was concluded that the CNN method, with prior data preparation, can be applied to this type of issue.

The accuracy assessment provides a truth versus visual interpretation of the overall classification. This is evidenced by the fact that both methods had problems in classifying the damage intensity index of the same buildings. When comparing the two methods, it is also important to note the low hardware requirements of the SVM when used to analyse the data in numerical form. However, no significant differences in performance between SVM and CNN were found during the conducted tests. Furthermore, as research^49^ shows, SVM can generate hardware capacity problems when analysing image data. The CNN may be disadvantaged by the additional time cost of converting numerical data into quasi-images, while on the other hand, it provides an opportunity to introduce additional information into the image and use it during analysis.

## Conclusions

The aim of the presented research was to create a model for the damage prediction of masonry residential buildings located in a mining area. In addition, the research discussed the procedure of preparing data for analysis, their preprocessing and implementation into the Convolutional Neural Network (CNN) method. A comparison was made between the CNN method and the Support Vector Machine (SVM) method, which is commonly used for analysis of this type of issue. This made it possible to evaluate the CNN method in terms of its suitability for this type of task.

Multiple analyses using different optimisation criteria were conducted as part of the research. For the CNN, the best-fitting network was the one using the SGDM (Stochastic Gradient Descent with Momentum) algorithm, which for the test set achieved a fitting accuracy *ACC* = 78.26%. A similar result was obtained for the training set (*ACC* = 78.42%), indicating the high generalisation ability of the model.

An SVM model was created for the comparisons. Its parameters were determined using Bayesian optimisation. Radial basis functions (RBF) were used as the kernel function of the model. The number of support vectors reduced to 90 relatives to the training set size (139) demonstrates the high generalisation properties of the SVM model. This was also confirmed by the small difference in fitting accuracy between the training set (*ACC* = 80.58) and the test set (*ACC* = 78.26).

Comparisons of the obtained models were made by examining parameters such as *ACC*, precision *PPV*, recall* TPR*, and* F*_1_ score. The comparisons revealed only minor differences between the models. The differences favoured the SVM model for training set accuracy, while they favoured the CNN model for *F*_1_ score and average *PPV* for the test set (Table 8). The detailed results for the individual categories were also similar. Therefore, it is not possible to conclude unequivocally that either model proved to be superior. On the one hand, the CNN model requires additional data preparation, which can be labour-intensive in the initial phase of the study. However, its application offers the hope of automating the damage prediction process for buildings. To this end, image analysis for damage assessment of individual building elements and entire buildings should be identified as a future research direction. This will reduce the human factor in assessing damage intensity to only taking photographs of damage and building a single model that is able to both assess the damage intensity of a building and make damage predictions, based on structural and material data, maintenance quality, and mining impacts.

It should be noted that the proposed models were used for the prediction of negligible damage, where the model distinguishes between 3 categories in the range of 0–6% damage intensity. This state of affairs allows for a detailed prediction of the damage intensity using methods from the classification. In addition, to obtain better results, the use of datasets with a larger number of buildings or/and parameters should be pursued. The main limitation of the applicability of the presented models is the small number of input data, which may reduce their potential.

The created models can now be used to predict the intensity of damage to buildings when data are available on the building and the impacts it has been subjected to so far. Considering the large number of buildings exposed to damage from mining impacts, this can lead to significant energy, economic, and environmental savings, as well as having a positive impact on users' standard of living.

## Data Availability

Data sets used during the current study are available upon reasonable request from the corresponding author.
